# Synthesis, crystal structure and Hirshfeld surface analysis of sulfamethoxazolium methyl­sulfate monohydrate

**DOI:** 10.1107/S2056989024009204

**Published:** 2024-09-24

**Authors:** Aldana B. Moroni, Tiago Bottoso, Diego F. Lionello, Daniel R. Vega, Teodoro S. Kaufman, Natalia L. Calvo

**Affiliations:** a Instituto de Química Rosario (IQUIR CONICET-UNR) and Facultad de Ciencias Bioquímicas y Farmacéuticas Universidad Nacional de Rosario. Suipacha 531 2000 Rosario Argentina; b Departamento Física de la Materia Condensada, Gerencia de Investigación y, Aplicaciones, Centro Atómico Constituyentes, Comisión Nacional de Energía, Atómica, Av. Gral. Paz 1499, B1650KNA, San Martín, Buenos Aires, Argentina; Universidade de Sâo Paulo, Brazil

**Keywords:** crystal structure, hydrogen bonding, methyl sulfate, mol­ecular salt, sulfamethoxazolium

## Abstract

The mol­ecular salt sulfamethoxazolium {or 4-[(5-methyl-1,2-oxazol-3-yl)sulf­amo­yl]anilinium methyl sulfate monohydrate}, C_10_H_12_N_3_O_3_S^+^·CH_3_O_4_S^−^·H_2_O, was prepared by the reaction of sulfamethoxazole and H_2_SO_4_ in methanol and crystallized from methanol–ether–water. Protonation takes place at the nitro­gen atom of the primary amino group. In the crystal, N—H⋯O hydrogen bonds (water and methyl­sulfate anion) and inter­molecular N—H⋯N inter­actions involving the sulfonamide and isoxazole nitro­gen atoms, link the components into a tri-dimensional network, additional cohesion being provided by face-to-face π–π inter­actions between the phenyl rings of adjacent mol­ecules. A Hirshfeld surface analysis was used to verify the contributions of the different inter­molecular inter­actions, showing that the three most important contributions for the crystal packing are from H⋯O (54.1%), H⋯H (29.2%) and H⋯N (5.0%) inter­actions.

## Abstract 2

The title mol­ecular salt was prepared by the reaction of sulfamethoxazole and H_2_SO_4_ in methanol and crystallized from methanol–ether–water. Protonation takes place at the nitro­gen atom of the primary amino group. In the crystal, N—H⋯O hydrogen bonds (water and methyl­sulfate anion) and inter­molecular N—H⋯N inter­actions involving the sulfonamide and isoxazole nitro­gen atoms, link the components into a tri-dimensional network, which also features face-to-face π–π inter­actions between the phenyl rings of adjacent mol­ecules.

## 1. Chemical context

Sulfamethoxazole {SMX or 4-[(5-methyl­isoxazol-3-yl)amino­sulfon­yl]aniline} is a widely employed sulfa drug that is effective against Gram-negative and Gram-positive bacteria, and active against some protozoans and fungi (Manyando *et al.*, 2013 ▸). Being structurally similar to *para*-amino­benzoic acid (PABA), it acts as a di­hydro­folate reductase inhibitor (Cushion & Walzer, 2009 ▸); it also competitively inhibits the enzyme di­hydro­pteroate synthase, preventing the biosynthesis of di­hydro­pteroic acid, a precursor of folic acid that is required for bacterial growth (Khalil *et al.*, 2003 ▸).

SMX has both low solubility and permeability; therefore, it is a Class IV drug in the Biopharmaceutical Classification System (BCS). The poor solubility of SMX has elicited continuous inter­est in finding alternative forms of the drug with improved pharmacological profiles. As a result, several polymorphs (Price *et al.*, 2005 ▸), hydrates (Alsubaie *et al.*, 2018 ▸; Takasuka & Nakai, 2001 ▸), metal complexes (Habila *et al.*, 2021 ▸), co-crystals [including that with trimethoprim (Bettinetti & Giordano, 1988 ▸), with which it forms a useful pharmaceutical association], and salts (de Moura Oliveira *et al.*, 2019 ▸) of SMX have been reported. In connection with our research program on the characterization of new solid phases derived from poorly soluble active pharmaceutical ingredients, herein we report on the crystal structure and the supra­molecular packing pattern of the acid methyl­sulfate monohydrate salt of SMX (SMXHMeSO_4_·H_2_O). Acid methyl­sulfate monohydrate salts of other active pharmaceutical ingredients have been reported (Gutiérrez *et al.*, 2020 ▸); among them is neostigmine methyl­sulfate, a cholinesterase inhibitor used in the treatment of myasthenia gravis and to reverse the effects of muscle relaxants (Papich, 2021 ▸) and pralidoxime methyl­sulfate, a widely agent used to treat organophosphate poisoning (Thompson *et al.*, 1987 ▸).
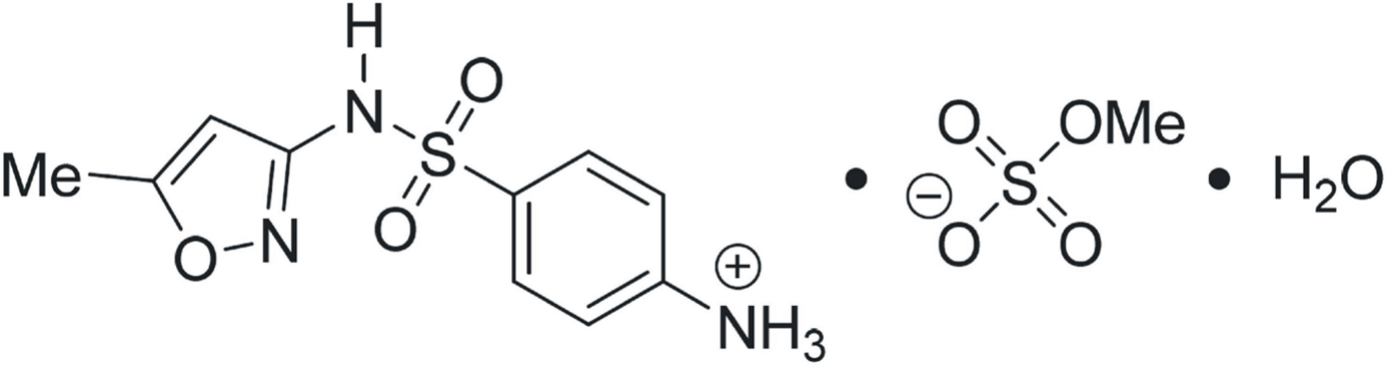


## 2. Structural commentary

**Figure 1:**
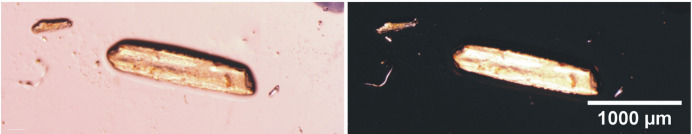
Microscopic view of a crystal of the title compound under white (left) and polarized (right) light.

**Figure 2:**
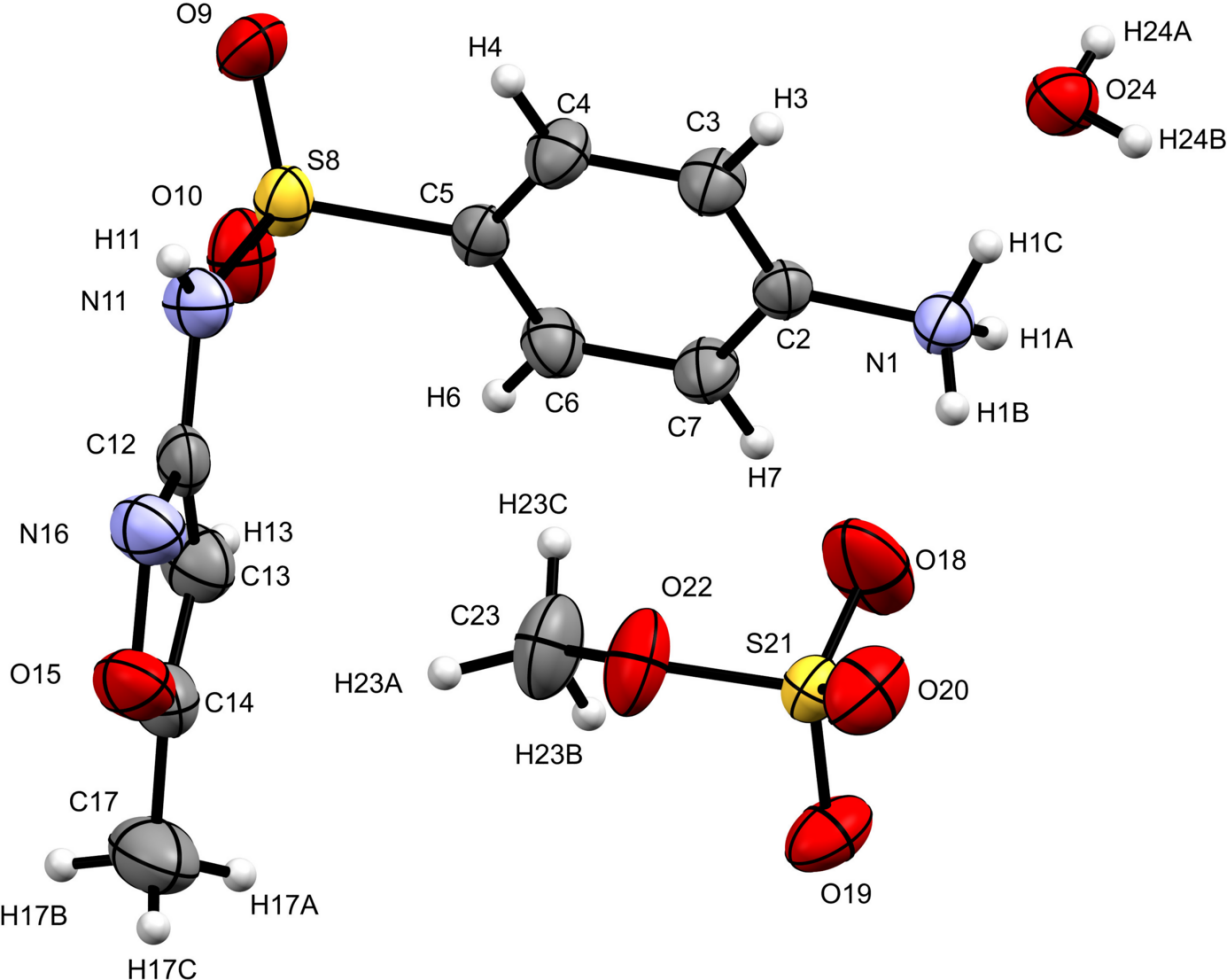
Crystal structure of title compound with the atom-labeling scheme (displacement ellipsoids are drawn at the 50% probability level).

The crystals of the title monohydrate salt SMXHMeSO_4_·H_2_O, (I), appear as small white rods under white light that display birefringence under polarized light (Fig. 1 ▸) and have a melting point 374.5–375.5 K. The title compound crystallizes in the triclinic space group *P*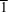 with one of each component (sulfamethoxazolium cation, methyl­sulfate anion, and water) in the asymmetric unit (Fig. 2 ▸).

The sulfamethoxazolium cation, which undergoes protonation at the primary amino nitro­gen, is L-shaped. The C6—C5—S8—N11 torsion angle is 96.4 (2)°, while the dihedral angle between the planes formed by the aromatic rings is 88.83 (13)° and the S8—N11—C12—N16 torsion angle is 162.5 (2)°.

**Figure 3:**
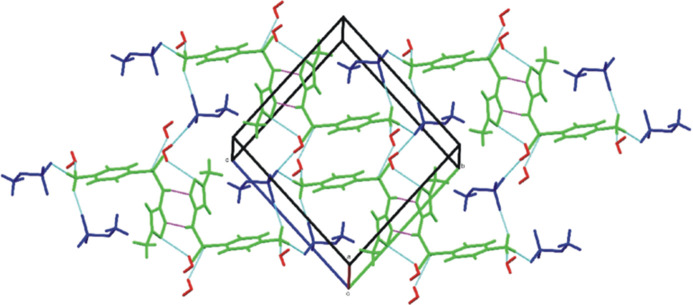
Crystal packing of the compound showing all the hydrogen-bonding inter­actions with an O atom as acceptor (cyan dashed lines) and the dimers formed by N—H⋯N inter­actions (magenta dashed lines).

The nitro­gen atom of the sulfonamide has *sp*^2^ character, as a result of conjugation; the S8—N11—C12 angle is 125.09 (17)° and the N—H moiety is almost coplanar with the isoxazole ring, with the H11—N11—C12—N16 torsion angle being −17°. The cation and the anion in the asymmetric unit are linked by the N1—H1*B*⋯O18 hydrogen bond (Fig. 3 ▸ and Table 1 ▸). The methyl group of the anion occupies the hydro­phobic pocket formed by the aromatic rings of the cation, with H23*C* located 2.96 Å from the centroid of the phenyl ring and H23*A* 2.81 Å from the centroid of the isoxazole ring. The N1—H1*C*⋯O24 hydrogen bond links the water mol­ecule to the cation. Both sulfur atoms exhibit slightly distorted tetra­hedral geometries; the O9—S8—O10 and O18—S21—O20 angles have values of 119.97 (12) and 115.06 (14)°, respectively, presenting the maximum deviations from the expected ones.

## 3. Supra­molecular features

The salt structure is consolidated by a three-dimensional network of hydrogen bonds between the anions, cations, and water mol­ecules, as well as by π–π inter­actions between aromatic rings (Fig. 3 ▸, Tables 1 ▸ and 2 ▸). Among the salient features, each water mol­ecule establishes hydrogen bonds with SMXH^+^ units, acting through O24 as an acceptor with H1*C* (protonated primary amino group, *x*, *y*, *z*) and through H24*A* as a donor with both, O10 (sulfonamide, −*x* + 1, −*y* + 1, −*z* + 1) and O15 (isoxazole, *x* − 1, *y*, *z* + 1) in a bifurcated inter­action. An additional hydrogen bond relates H24*B* with O19 of the methyl­sulfate anion (−*x*, −y, −*z* + 1).

Each methyl­sulfate anion establishes four inter­actions; two of them are hydrogen bonds O18⋯H1*B* (*x*, *y*, *z*) and O19⋯H1*A* (−*x* + 1, −*y*, −*z* + 1) with slightly different lengths (1.944 and 1.913 Å, respectively), that bridge a pair of adjacent SMX mol­ecules through their protonated amino moieties, while the third hydrogen bond is an inter­action with a water mol­ecule (O19⋯H24*B*). The fourth is a C—H⋯O inter­molecular inter­action between H23*B* and O20 of a neighboring methyl­sulfate anion (*x* + 1, *y*, *z*), which results in a chain of methyl­sulfate anions running along the *a*-axis direction.

In addition, the SMXH^+^ units are also directly connected through pairs of N11—H11⋯N16 hydrogen bonds (−*x* + 1, −*y* + 1, −*z*), which involve the isoxazole nitro­gen atom (N16) and the sulfonamide N—H moiety (N11—H11).

The structure also features face-to-face π–π inter­actions between the phenyl rings of adjacent mol­ecules, which adopt an anti­parallel arrangement, in parallel planes. In one of them (1 − *x*, 1 − *y*, 1 − *z*), the planes are 3.5674 (10) Å apart (Table 2 ▸), and the stacked aromatic rings are slipped by 2.535 Å. This aromatic ring displacement (slippage) is the distance between the perpendicular projection of the centroid of one ring on the other and the centroid of the latter. In addition, the mean slippage angle (sa, the angle subtended by the inter-centroid vector to the plane normal) is 35.4°, whereas the distance between centroids is 4.3764 (14) Å. Face-to-face π–π inter­actions are also observed between the isoxazole rings (2 − *x*, 1 − *y*, −*z*), which are also arranged in an anti­parallel fashion. The inter­planar distance is 3.5028 (10) Å while the inter­centroid distance is 4.8490 (16) Å, resulting in a slippage angle of 43.7° and a slippage of 3.353 Å. Considering the geometrical requirements for inter­actions between aromatic rings (Hunter & Sanders, 1990 ▸), the parameters of both slipped packings correspond to attractive inter­actions.

The π–π inter­actions between aromatic rings play an important role in controlling the packing or assembly of mol­ecules. Usually, they take the form of an offset or slipped stacking, where the rings are parallel displaced a certain distance (slippage, aromatic ring displacement). These inter­actions between aromatic rings of adjacent mol­ecules seem to be one of the characteristic features of the sulfamethoxazolium derivatives, being found in several congeners of the title compound (Table 2 ▸).

The formation of anti­parallel π-stacking inter­actions in these compounds may contribute to the cohesion of the crystal, considering that the phenyl ring has an electron-poor region at the sulfonamide side, opposite to a more electron-rich zone on the protonated amino region. In addition, the positively charged atom contributes to the attractive π–σ inter­action due to the induced σ polarization.

## 4. Hirshfeld surface analysis

**Figure 4:**
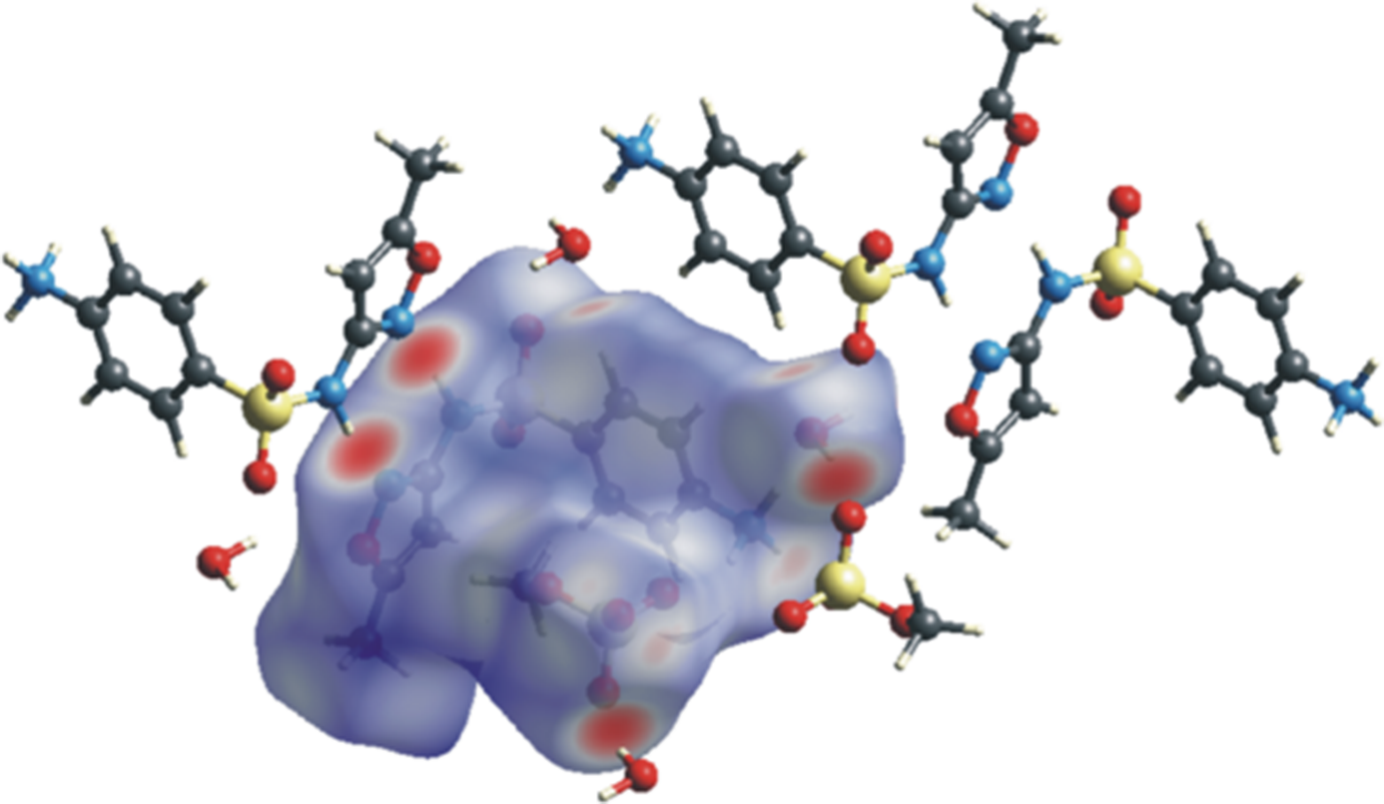
View of the three-dimensional Hirshfeld surface of the title mol­ecule plotted over *d*_norm_.

The three-dimensional Hirshfeld surface (McKinnon *et al.*, 2007 ▸) with a *d*_norm_ (normalized contact distance) plot (Fig. 4 ▸) and two-dimensional fingerprint plots (Spackman & McKinnon, 2002 ▸) were generated with *Crystal Explorer 17.5* (Spackman *et al.*, 2021 ▸). This analysis was carried out to verify the presence of inter­molecular inter­actions and hydrogen bonds in the crystal structure and assess the contributions from the different inter­molecular inter­actions in the title compound.

The Hirshfeld surface was plotted over the range −0.6318 (red) to +1.4441 (blue) a.u. The red spots on the top left of the surface indicate the sites of the N11—H11⋯N16 inter­actions (−*x* + 1, −*y* + 1, −*z*) between the sulfonamide N—H moiety and the nitro­gen atom of the isoxazole, while at the top right, the place of the O⋯H inter­action between the sulfonamide and the isoxazole with water can be observed.

**Figure 5:**
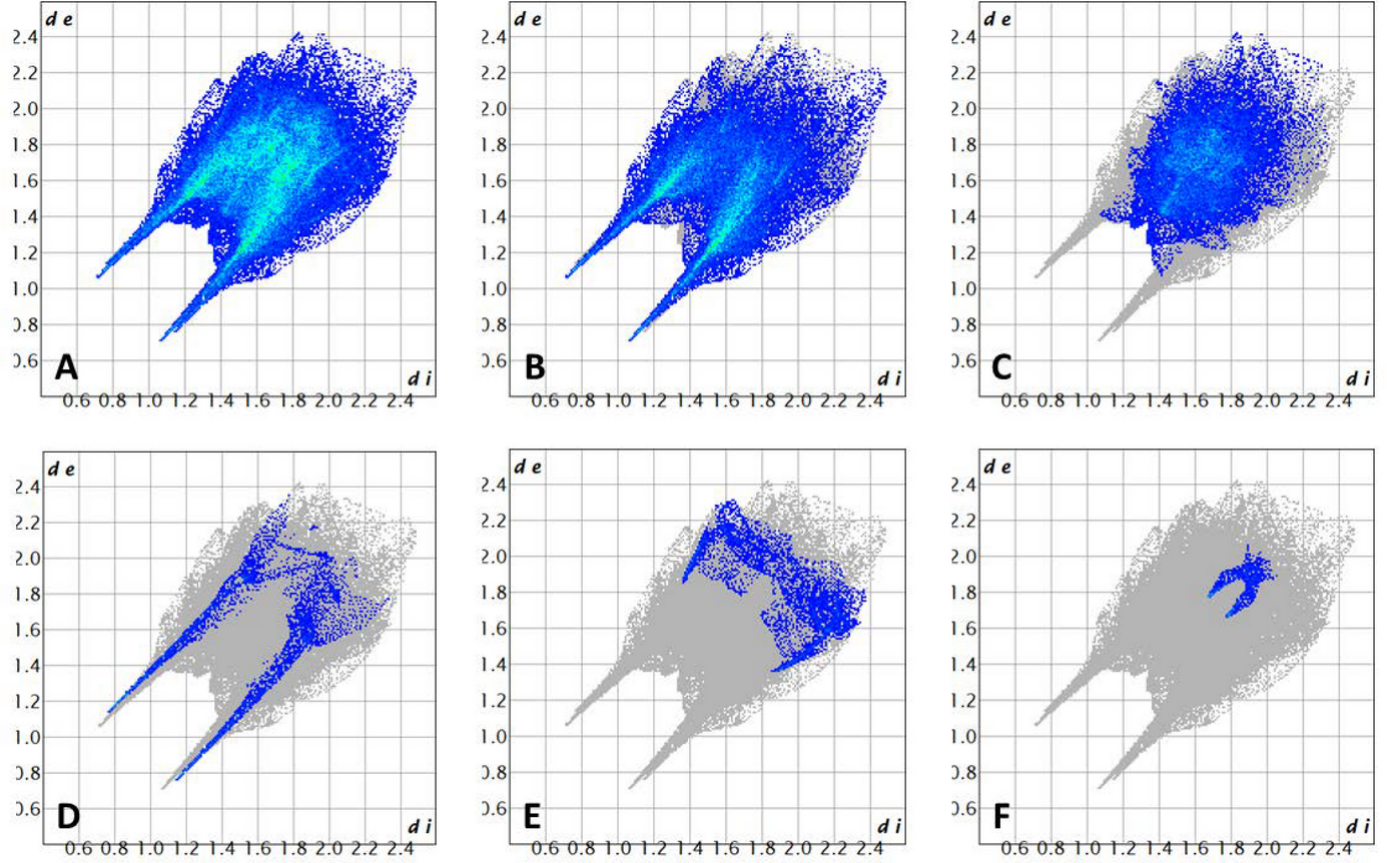
Two-dimensional fingerprint plots for the title mol­ecule showing (A) all inter­actions, and those delineated into (B) H⋯O/O⋯H inter­actions, (C) H⋯H inter­actions, (D) H⋯N/N⋯H inter­actions (E) H⋯C/C⋯H inter­actions, and (F) C⋯N/N⋯C inter­actions. The *d_i_* and *d_e_* values are the closest inter­nal and external distances (in Å) from given points on the Hirshfeld surface.

A C4—H4⋯O24 (*x*, *y*, *z*) inter­action site with water is also visible on top. In addition, the sites of inter­action of H_2_O with oxygen atoms of the neighbouring methyl­sulfate anion O24—H24*B*⋯O19 (−*x*, −*y*, −*z* + 1) are shown on the top right and the same inter­action can be observed at the bottom right. The 2D fingerprint plots (Fig. 5 ▸) revealed that the greatest contributions to the total inter­molecular inter­actions (Fig. 5 ▸A) are from H⋯O/O⋯H contacts (54.1%), which appear in the middle of the scattered points of the 2D fingerprint plot, along with two symmetrical broad wings (Fig. 5 ▸B), followed by H⋯H contacts observed in the middle of the scattered points in the plot (29.2%, Fig. 5 ▸C), and H⋯N/N⋯H contacts (5.0%, Fig. 5 ▸D), which result from the inter­actions between the sulfonamide N—H moiety and the nitro­gen atom of the isoxazole to form a dimer, and are present as sharp symmetrical spikes at diagonal axes.

The proportions of these contributions are the expected ones due to the significant hydrogen content, which is present in the three components of the salt, and the fact that many of them are attached to heteroatoms. These inter­actions suggest that hydrogen bonding plays a major role in the crystal packing. The contributions to the Hirshfeld surface from other inter­atomic inter­actions include H⋯C/C⋯H, which are displayed as bump symmetrical spikes at diagonal axes (5.0%, Fig. 5 ▸E), C⋯O/O⋯C (2.6%) and C⋯N/N⋯C contacts (1.0%, Fig. 5 ▸F). In comparison, N⋯O/O⋯N (1.1%), C⋯S/S⋯C (0.1%), and H⋯S/S⋯H (0.1%) contacts represent additional, minor participations.

## 5. Database survey

A simple search in the Cambridge Structural Database (CSD, accessed *via* WebCSD on September 19, 2024; Groom *et al.*, 2016 ▸) with the keyword ‘sulfa­methoxazole’ gave 73 hits, of which only six involved the sulfamethoxazolium ion, and included the following salts: chloride (SIMJEE, Subashini *et al.*, 2007 ▸), bromide (GAGLAS, de Moura Oliveira *et al.*, 2019 ▸), nitrate (GOGLEW, de Moura Oliveira *et al.*, 2019 ▸), penta­iodide monohydrate (CIDDAY, de Moura Oliveira *et al.*, 2019 ▸), 3,5-di­nitro­salicylate (TUJPEV, Malathy *et al.*, 2015 ▸), and the metallic complex *catena*-[bis­(sulfa­methox­azo­lium)(μ_2_-chlor­ido­tri­chlorido­cadmium(II) monohydrate] (RISZAV, Subashini *et al.*, 2008 ▸). A more in-depth search of the database, using the keyword ‘sulfamethoxazolium’ uncovered the metallic complex tri­chloro-{4-[(5-methyl-1,2-oxazol-3-yl)sulfamo­yl]anilinium}zinc (AWARIC, Habila *et al.*, 2021 ▸) as the seventh member of this family of compounds.

In all cases, the structure of the protonated form of SMX is L-shaped, displaying dihedral angles between the mean planes of the phenyl ring and the isoxazole unit of 58° (SIMJEE), 75° (GAGLAS), 87° (GOGLEW), 87° (CIDDAY), 82° (TUJPEV), 88.3° (RISZAV) and 89.2° (AWARIC). The simple halide salts (chloride and bromide) displayed the smallest values for the dihedral angle between the planes containing the isoxazole and anilinium rings. The S8—N11—C12—N16 torsion angles of the compounds presented the following values: −57.2 (4)° (SIMJEE), −25.3 (4)° (GAGLAS), −26.38 (1)° (CIDDAY), −152.4 (4)/151.5 (4)° (GOGLEW), −164.3 (2)° (AWARIC), 164.49 (14)° (TUJPEV) and 158.6 (3)° (RISZAV). Accordingly, salts in this series could be grouped in two sets; on one side the halides, with a small torsion angle, measuring less than 60°, and the remaining compounds including SMXHMeSO_4_·H_2_O on the other, with large torsions, where the angles are above 150°. This suggests that the isoxazole ring in these compounds adopts two main orientations.

## 6. Synthesis and crystallization

A sample of sulfamethoxazole (250 mg, 0.99 mmol) was dissolved in methanol (25 mL) at room temperature and treated with a methano­lic solution of H_2_SO_4_ (0.507 *M*, 1.95 mL, 0.99 mmol). After stirring for 30 min, the solution was allowed to concentrate to 12 mL (3 days) at room temperature, when Et_2_O (54 mL) containing water (0.1%, 0.054 mL, 3 mmol) was added. The system was left to stand at room temperature and crystals (15 mg) were collected after 6 d.

## 7. Refinement

Table 3 ▸ summarizes crystal data, data collection, and structure refinement details. The H atoms were positioned geometrically and refined using a riding model: O—H = 0.82 Å, N—H = 0.86–0.89 Å, and C—H = 0.93–0.96 Å with *U*_iso_(H) = 1.5*U*_eq_(C,O,N) for methyl and ammonium H atoms and 1.2*U*_eq_(C,N) for aromatic and other H atoms. Water H atoms were found in difference-Fourier maps and refined independently.

## Supplementary Material

Crystal structure: contains datablock(s) I. DOI: 10.1107/S2056989024009204/ex2088sup1.cif

Structure factors: contains datablock(s) I. DOI: 10.1107/S2056989024009204/ex2088Isup2.hkl

Supporting information file. DOI: 10.1107/S2056989024009204/ex2088Isup3.cml

CCDC reference: 2385280

Additional supporting information:  crystallographic information; 3D view; checkCIF report

## Figures and Tables

**Table 1:** Hydrogen-bond geometry (Å, °)

*D*—H⋯*A*	*D*—H	H⋯*A*	*D*⋯*A*	*D*—H⋯*A*
C3—H3⋯O24	0.93	2.65	3.353 (3)	133
C4—H4⋯O24^i^	0.93	2.54	3.469 (3)	177
C13—H13⋯O10	0.93	2.54	3.023 (3)	113
N1—H1*A*⋯O19^ii^	0.89	1.91	2.800 (3)	175
N1—H1*A*⋯S21^ii^	0.89	2.94	3.774 (2)	156
N1—H1*B*⋯O18	0.89	1.95	2.761 (3)	152
N1—H1*C*⋯O24	0.89	1.89	2.772 (3)	170
N11—H11⋯N16^iii^	0.86	2.07	2.912 (3)	167
C23—H23*B*⋯O20^iv^	0.96	2.65	3.506 (4)	149
O24—H24*A*⋯O10^v^	0.76 (3)	2.54 (3)	3.140 (3)	137 (3)
O24—H24*A*⋯O15^vi^	0.76 (3)	2.47 (3)	3.072 (3)	137 (3)
O24—H24*B*⋯O19^vii^	0.81 (3)	2.03 (3)	2.821 (3)	163 (3)

Symmetry codes: (i) 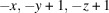; (ii) 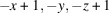; (iii) 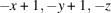; (iv) 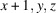; (v) 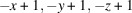; (vi) 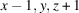; (vii) 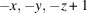.

**Table 2:** Selected details of π–π inter­actions (Å, °) for some sulfamethoxazolium salts Structures with centroid–centroid separations < 6.0 Å and α < 20.00° according to *PLATON* (Spek, 2020 ▸). *Cg*1, *Cg*2, *Cg*3 and *Cg*4 are the centroids of the O15/N16/C12–C14, C2–C7, O15′/N16′/C12′–C14′ and C2′–C7′ rings, respectively. α is the dihedral angle between planes *I* and *J*; ccd is the distance between ring centroids, ipd is the mean inter­planar distance (distance from one plane to the neighboring centroid), slippage is distance between *Cg*(*I*) and the perpendicular projection of *Cg*(*J*) on ring *I* and sa is the mean slippage angle (angle subtended by the inter-centroid vector to the plane normal). For additional details, see Janiak (2000 ▸).

Refcode	*Cg*(*I*)⋯*Cg*(*J*)	α	ccd	ipd	slippage	sa
(I)	*Cg*1⋯*Cg*1^i^	0.02 (14)	4.8490 (16)	3.5028 (10)	3.353	43.7
	*Cg*2⋯*Cg*2^ii^	0.03 (11)	5.8838 (16)	3.0064 (10)	5.058	59.3
	*Cg*2⋯*Cg*2^iii^	0.03 (11)	4.3764 (14)	3.5674 (10)	2.535	35.4
CIDDAY	*Cg*2⋯*Cg*2^iv^	0	3.8532	3.5757	1.436	21.9
TUJPEV	*Cg*2⋯*Cg*2^v^	0	5.9284	3.3032	4.923	56.1
GAGLAS	*Cg*1⋯*Cg*1^vi^	17	5.4016	3.4095	4.190	50.9
	*Cg*1⋯*Cg*2^vii^	0	4.2924	3.8496	1.899	26.3
RISZAV	*Cg*1⋯*Cg*1^viii^	17	4.7430	3.8184	2.813	36.4
	*Cg*1⋯*Cg*1i^*x*^	17	4.7430	2.9750	3.694	51.2
	*Cg*1⋯*Cg*1^*x*^	0	4.4550	3.2790	3.016	42.6
	*Cg*2⋯*Cg*2^xi^	0	5.5620	3.0159	4.673	57.2
GOGLEW	*Cg*1/*Cg*3^xii^	0	3.8495	3.3844	1.834	28.5
	*Cg*1⋯*Cg*3^xiii^	0	3.9641	3.2582	2.258	34.7
	*Cg*2⋯*Cg*2^iii^	0	5.3927	3.0874	4.421	55.1
	*Cg*4⋯*Cg*4^xiv^	0	5.3927	3.0831	4.424	55.1
AWARIC	*Cg*1⋯*Cg*1^xv^	0	4.3792	4.1868	1.283	17.0
	*Cg*2⋯*Cg*2^xvi^	0	4.1198	3.4480	2.255	33.2

Symmetry codes: (i) 2 − *x*, 1 − *y*, −*z*; (ii) −1 + *x*, *y*, *z*; (iii) 1 − *x*, 1 − *y*, 1 − *z*; (iv) 1 − *x*, 2 − *y*, 1 − *z*; (v) 1 − *x*, 2 − *y*, 1 − *z*; (vi) 1 − *x*, *y*, 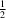 − *z*; (vii) 1 − *x*, −*y*, −*z*; (viii) 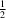 − *x*, −*y*, −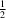 + *z*; (ix) 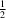 − *x*, −*y*, 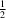 + *z*; (*x*) 1 − *x*, −*y*, 2 − *z*; (*x*i) *x*, *y*, −1 + *z*; (xii) 1 − *x*, 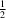 + *y*, 1 − *z*; (xiii) 1 − *x*, 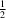 + *y*, 2 − *z*; (xiv) 1 − *x*, *y*, *z*; (xv) 2 − *x*, −*y*, −*z*; (xvi) 1 − *x*, −*y*, −1 − *z*.

**Table 3:** Experimental details

Crystal data	
Chemical formula	C_10_H_12_N_3_O_3_S^+^·CH_3_O_4_S^−^·H_2_O
*M*_r_	383.39
Crystal system, space group	Triclinic, *P* 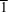
Temperature (K)	298
*a*, *b*, *c* (Å)	5.8838 (8), 11.6532 (15), 12.3276 (16)
α, β, γ (°)	84.722 (6), 78.442 (5), 81.940 (5)
*V* (Å^3^)	818.14 (19)
*Z*	2
Radiation type	Mo *K*α
μ (mm^−1^)	0.37
Crystal size (mm)	1.00 × 0.35 × 0.12

Data collection	
Diffractometer	Bruker D8 Quest ECO
Absorption correction	Multi-scan (*SADABS*; Krause *et al.*, 2015 ▸)
*T*_min_, *T*_max_	0.854, 0.956
No. of measured, independent and observed [*I* > 2σ(*I*)] reflections	18938, 3318, 2716
*R*_int_	0.047
(sin θ/λ)_max_ (Å^−1^)	0.625

Refinement	
*R*[*F*^2^ > 2σ(*F*^2^)], *wR*(*F*^2^), *S*	0.043, 0.100, 1.06
No. of reflections	3318
No. of parameters	226
H-atom treatment	H atoms treated by a mixture of independent and constrained refinement
Δρ_max_, Δρ_min_ (e Å^−3^)	0.33, −0.32

Computer programs: *APEX4* and *SAINT* (Bruker, 2019 ▸), *SHELXS97* (Sheldrick, 2008 ▸), *SHELXL2019/1* (Sheldrick, 2015 ▸), *POV-RAY* (Cason, 2004 ▸), *Mercury* (Macrae *et al.*, 2020 ▸), *PLATON* (Spek, 2020 ▸), *publCIF* (Westrip, 2010 ▸) and *WinGX* (Farrugia, 2012 ▸).

## supplementary crystallographic information

### 4-[(5-Methyl-1,2-oxazol-3-yl)sulfamoyl]anilinium methyl sulfate monohydrate Crystal data

**Table:**

C_10_H_12_N_3_O_3_S^+^·CH_3_O_4_S^−^·H_2_O	*Z* = 2
*M**_r_* = 383.39	*F*(000) = 400
Triclinic, *P*1	*D*_x_ = 1.556 Mg m^−^^3^
*a* = 5.8838 (8) Å	Mo *K*α radiation, λ = 0.71073 Å
*b* = 11.6532 (15) Å	Cell parameters from 1942 reflections
*c* = 12.3276 (16) Å	θ = 2.3–26.2°
α = 84.722 (6)°	µ = 0.37 mm^−^^1^
β = 78.442 (5)°	*T* = 298 K
γ = 81.940 (5)°	Needle, colourless
*V* = 818.14 (19) Å^3^	1.00 × 0.35 × 0.12 mm

### 4-[(5-Methyl-1,2-oxazol-3-yl)sulfamoyl]anilinium methyl sulfate monohydrate Data collection

**Table:**

Bruker D8 Quest ECO diffractometer	2716 reflections with *I* > 2σ(*I*)
Radiation source: Sealed tube	*R*_int_ = 0.047
ω scan	θ_max_ = 26.4°, θ_min_ = 3.4°
Absorption correction: multi-scan (SADABS; Krause *et al.*, 2015)	*h* = −7→7
*T*_min_ = 0.854, *T*_max_ = 0.956	*k* = −14→14
18938 measured reflections	*l* = −15→15
3318 independent reflections	

### 4-[(5-Methyl-1,2-oxazol-3-yl)sulfamoyl]anilinium methyl sulfate monohydrate Refinement

**Table:**

Refinement on *F*^2^	0 restraints
Least-squares matrix: full	Hydrogen site location: mixed
*R*[*F*^2^ > 2σ(*F*^2^)] = 0.043	H atoms treated by a mixture of independent and constrained refinement
*wR*(*F*^2^) = 0.100	*w* = 1/[σ^2^(*F*_o_^2^) + (0.0142*P*)^2^ + 1.0486*P*] where *P* = (*F*_o_^2^ + 2*F*_c_^2^)/3
*S* = 1.06	(Δ/σ)_max_ < 0.001
3318 reflections	Δρ_max_ = 0.33 e Å^−^^3^
226 parameters	Δρ_min_ = −0.32 e Å^−^^3^

### 4-[(5-Methyl-1,2-oxazol-3-yl)sulfamoyl]anilinium methyl sulfate monohydrate Special details

**Table:**

Geometry. All esds (except the esd in the dihedral angle between two l.s. planes) are estimated using the full covariance matrix. The cell esds are taken into account individually in the estimation of esds in distances, angles and torsion angles; correlations between esds in cell parameters are only used when they are defined by crystal symmetry. An approximate (isotropic) treatment of cell esds is used for estimating esds involving l.s. planes.

### 4-[(5-Methyl-1,2-oxazol-3-yl)sulfamoyl]anilinium methyl sulfate monohydrate Fractional atomic coordinates and isotropic or equivalent isotropic displacement parameters (Å^2^)

**Table:**

	*x*	*y*	*z*	*U*_iso_*/*U*_eq_	
C3	0.3075 (4)	0.3673 (2)	0.4655 (2)	0.0359 (5)	
H3	0.152494	0.384591	0.500421	0.043*	
C4	0.4132 (4)	0.4451 (2)	0.3856 (2)	0.0375 (5)	
H4	0.330192	0.515477	0.366393	0.045*	
C6	0.7715 (4)	0.3127 (2)	0.3633 (2)	0.0368 (5)	
H6	0.926915	0.295236	0.329032	0.044*	
C7	0.6653 (4)	0.2354 (2)	0.4430 (2)	0.0367 (5)	
H7	0.748077	0.165273	0.462965	0.044*	
C13	0.9869 (5)	0.3140 (2)	0.0578 (2)	0.0475 (6)	
H13	1.096502	0.312564	0.102944	0.057*	
N1	0.3214 (3)	0.18050 (16)	0.57383 (16)	0.0334 (4)	
H1A	0.418354	0.149870	0.618726	0.050*	
H1B	0.284132	0.124328	0.538887	0.050*	
H1C	0.192294	0.216536	0.613491	0.050*	
C2	0.4356 (4)	0.26394 (19)	0.49225 (18)	0.0293 (5)	
C5	0.6438 (4)	0.41640 (19)	0.33507 (18)	0.0301 (5)	
S8	0.77327 (11)	0.51495 (5)	0.23174 (5)	0.03585 (16)	
O9	0.6651 (4)	0.63011 (15)	0.25416 (15)	0.0480 (5)	
O10	1.0205 (3)	0.48770 (17)	0.21978 (15)	0.0489 (5)	
N11	0.6972 (4)	0.48975 (18)	0.11723 (16)	0.0389 (5)	
H11	0.595794	0.540280	0.092239	0.047*	
C12	0.7835 (4)	0.3928 (2)	0.05656 (18)	0.0357 (5)	
C14	0.9881 (5)	0.2412 (2)	−0.0204 (2)	0.0488 (7)	
O15	0.7978 (4)	0.27161 (17)	−0.06734 (15)	0.0530 (5)	
N16	0.6641 (4)	0.36926 (19)	−0.01609 (18)	0.0452 (5)	
C17	1.1508 (7)	0.1406 (3)	−0.0656 (3)	0.0721 (10)	
H17A	1.196861	0.090255	−0.005591	0.108*	
H17B	1.286665	0.167607	−0.112248	0.108*	
H17C	1.074268	0.098765	−0.108343	0.108*	
O18	0.2313 (5)	0.0622 (2)	0.40614 (18)	0.0788 (8)	
O19	0.3568 (3)	−0.09508 (15)	0.28979 (18)	0.0516 (5)	
O20	−0.0403 (3)	−0.00330 (17)	0.30985 (18)	0.0548 (5)	
S21	0.19689 (10)	0.01013 (5)	0.31111 (5)	0.03422 (16)	
O22	0.2620 (4)	0.09783 (18)	0.20879 (18)	0.0594 (6)	
C23	0.4913 (6)	0.1348 (3)	0.1905 (3)	0.0656 (9)	
H23A	0.538503	0.158159	0.113494	0.098*	
H23B	0.600925	0.071669	0.211404	0.098*	
H23C	0.487101	0.199088	0.234604	0.098*	
O24	−0.1063 (3)	0.28881 (17)	0.67780 (16)	0.0431 (4)	
H24A	−0.102 (5)	0.317 (3)	0.730 (3)	0.052*	
H24B	−0.199 (5)	0.242 (3)	0.696 (2)	0.052*	

### 4-[(5-Methyl-1,2-oxazol-3-yl)sulfamoyl]anilinium methyl sulfate monohydrate Atomic displacement parameters (Å^2^)

**Table:**

	*U*^11^	*U*^22^	*U*^33^	*U*^12^	*U*^13^	*U*^23^
C3	0.0297 (11)	0.0353 (12)	0.0408 (13)	−0.0011 (9)	−0.0058 (10)	0.0009 (10)
C4	0.0379 (13)	0.0318 (12)	0.0416 (13)	−0.0005 (10)	−0.0101 (11)	0.0037 (10)
C6	0.0303 (12)	0.0414 (13)	0.0354 (12)	−0.0026 (10)	−0.0007 (10)	0.0000 (10)
C7	0.0348 (12)	0.0320 (12)	0.0390 (13)	0.0026 (10)	−0.0031 (10)	0.0021 (10)
C13	0.0503 (16)	0.0525 (16)	0.0381 (14)	0.0032 (13)	−0.0121 (12)	−0.0013 (12)
N1	0.0324 (10)	0.0298 (10)	0.0361 (10)	−0.0043 (8)	−0.0029 (8)	0.0005 (8)
C2	0.0331 (11)	0.0284 (11)	0.0277 (11)	−0.0075 (9)	−0.0062 (9)	−0.0021 (8)
C5	0.0359 (12)	0.0309 (11)	0.0262 (11)	−0.0089 (9)	−0.0101 (9)	−0.0003 (9)
S8	0.0424 (3)	0.0375 (3)	0.0309 (3)	−0.0151 (3)	−0.0095 (2)	0.0020 (2)
O9	0.0693 (13)	0.0306 (9)	0.0461 (10)	−0.0144 (8)	−0.0106 (9)	−0.0014 (8)
O10	0.0410 (10)	0.0654 (12)	0.0441 (10)	−0.0233 (9)	−0.0105 (8)	0.0070 (9)
N11	0.0472 (12)	0.0394 (11)	0.0315 (10)	−0.0008 (9)	−0.0142 (9)	−0.0012 (8)
C12	0.0431 (13)	0.0385 (13)	0.0244 (11)	−0.0097 (10)	−0.0045 (10)	0.0059 (9)
C14	0.0604 (17)	0.0471 (15)	0.0342 (13)	−0.0007 (13)	−0.0039 (12)	0.0015 (11)
O15	0.0699 (13)	0.0505 (11)	0.0399 (10)	−0.0009 (10)	−0.0138 (9)	−0.0115 (9)
N16	0.0529 (13)	0.0470 (13)	0.0366 (11)	−0.0016 (10)	−0.0116 (10)	−0.0070 (10)
C17	0.087 (3)	0.063 (2)	0.0548 (19)	0.0158 (18)	−0.0010 (17)	−0.0110 (16)
O18	0.0975 (18)	0.1011 (19)	0.0486 (12)	−0.0523 (15)	−0.0010 (12)	−0.0284 (12)
O19	0.0400 (10)	0.0333 (9)	0.0801 (14)	0.0002 (8)	−0.0135 (10)	0.0011 (9)
O20	0.0323 (10)	0.0514 (11)	0.0801 (15)	−0.0074 (8)	−0.0105 (9)	0.0018 (10)
S21	0.0355 (3)	0.0317 (3)	0.0360 (3)	−0.0073 (2)	−0.0072 (2)	0.0005 (2)
O22	0.0530 (12)	0.0613 (13)	0.0628 (13)	−0.0149 (10)	−0.0189 (10)	0.0302 (10)
C23	0.0603 (19)	0.066 (2)	0.069 (2)	−0.0264 (16)	−0.0070 (16)	0.0193 (16)
O24	0.0424 (10)	0.0425 (11)	0.0451 (11)	−0.0108 (8)	−0.0041 (9)	−0.0080 (8)

### 4-[(5-Methyl-1,2-oxazol-3-yl)sulfamoyl]anilinium methyl sulfate monohydrate Geometric parameters (Å, º)

**Table:**

C3—C2	1.378 (3)	S8—N11	1.626 (2)
C3—C4	1.388 (3)	N11—C12	1.391 (3)
C3—H3	0.9300	N11—H11	0.8600
C4—C5	1.384 (3)	C12—N16	1.312 (3)
C4—H4	0.9300	C14—O15	1.351 (3)
C6—C5	1.385 (3)	C14—C17	1.481 (4)
C6—C7	1.385 (3)	O15—N16	1.412 (3)
C6—H6	0.9300	C17—H17A	0.9600
C7—C2	1.374 (3)	C17—H17B	0.9600
C7—H7	0.9300	C17—H17C	0.9600
C13—C14	1.340 (4)	O18—S21	1.429 (2)
C13—C12	1.405 (4)	O19—S21	1.4472 (18)
C13—H13	0.9300	O20—S21	1.4291 (18)
N1—C2	1.463 (3)	S21—O22	1.5689 (19)
N1—H1A	0.8900	O22—C23	1.442 (3)
N1—H1B	0.8900	C23—H23A	0.9600
N1—H1C	0.8900	C23—H23B	0.9600
C5—S8	1.763 (2)	C23—H23C	0.9600
S8—O10	1.4253 (19)	O24—H24A	0.76 (3)
S8—O9	1.4285 (19)	O24—H24B	0.81 (3)
			
C2—C3—C4	119.0 (2)	N11—S8—C5	106.22 (10)
C2—C3—H3	120.5	C12—N11—S8	125.09 (17)
C4—C3—H3	120.5	C12—N11—H11	117.5
C5—C4—C3	119.0 (2)	S8—N11—H11	117.5
C5—C4—H4	120.5	N16—C12—N11	117.4 (2)
C3—C4—H4	120.5	N16—C12—C13	112.3 (2)
C5—C6—C7	119.3 (2)	N11—C12—C13	130.3 (2)
C5—C6—H6	120.4	C13—C14—O15	109.8 (2)
C7—C6—H6	120.4	C13—C14—C17	134.3 (3)
C2—C7—C6	118.9 (2)	O15—C14—C17	115.9 (3)
C2—C7—H7	120.5	C14—O15—N16	108.66 (19)
C6—C7—H7	120.5	C12—N16—O15	104.5 (2)
C14—C13—C12	104.7 (2)	C14—C17—H17A	109.5
C14—C13—H13	127.6	C14—C17—H17B	109.5
C12—C13—H13	127.6	H17A—C17—H17B	109.5
C2—N1—H1A	109.5	C14—C17—H17C	109.5
C2—N1—H1B	109.5	H17A—C17—H17C	109.5
H1A—N1—H1B	109.5	H17B—C17—H17C	109.5
C2—N1—H1C	109.5	O18—S21—O20	115.06 (14)
H1A—N1—H1C	109.5	O18—S21—O19	111.33 (15)
H1B—N1—H1C	109.5	O20—S21—O19	113.41 (11)
C7—C2—C3	122.3 (2)	O18—S21—O22	106.60 (14)
C7—C2—N1	118.9 (2)	O20—S21—O22	103.29 (12)
C3—C2—N1	118.9 (2)	O19—S21—O22	106.17 (12)
C4—C5—C6	121.5 (2)	C23—O22—S21	117.03 (18)
C4—C5—S8	118.33 (17)	O22—C23—H23A	109.5
C6—C5—S8	120.15 (18)	O22—C23—H23B	109.5
O10—S8—O9	119.97 (12)	H23A—C23—H23B	109.5
O10—S8—N11	108.68 (11)	O22—C23—H23C	109.5
O9—S8—N11	104.56 (11)	H23A—C23—H23C	109.5
O10—S8—C5	107.58 (11)	H23B—C23—H23C	109.5
O9—S8—C5	109.04 (11)	H24A—O24—H24B	106 (3)
			
C2—C3—C4—C5	0.2 (4)	O9—S8—N11—C12	172.3 (2)
C5—C6—C7—C2	−0.1 (4)	C5—S8—N11—C12	−72.5 (2)
C6—C7—C2—C3	−0.4 (4)	S8—N11—C12—N16	162.55 (18)
C6—C7—C2—N1	178.0 (2)	S8—N11—C12—C13	−19.1 (4)
C4—C3—C2—C7	0.4 (4)	C14—C13—C12—N16	1.1 (3)
C4—C3—C2—N1	−178.0 (2)	C14—C13—C12—N11	−177.3 (2)
C3—C4—C5—C6	−0.7 (4)	C12—C13—C14—O15	−0.4 (3)
C3—C4—C5—S8	178.67 (18)	C12—C13—C14—C17	178.0 (3)
C7—C6—C5—C4	0.7 (4)	C13—C14—O15—N16	−0.4 (3)
C7—C6—C5—S8	−178.69 (18)	C17—C14—O15—N16	−179.2 (2)
C4—C5—S8—O10	160.72 (18)	N11—C12—N16—O15	177.28 (19)
C6—C5—S8—O10	−19.9 (2)	C13—C12—N16—O15	−1.4 (3)
C4—C5—S8—O9	29.1 (2)	C14—O15—N16—C12	1.1 (3)
C6—C5—S8—O9	−151.45 (19)	O18—S21—O22—C23	55.1 (3)
C4—C5—S8—N11	−83.0 (2)	O20—S21—O22—C23	176.7 (2)
C6—C5—S8—N11	96.4 (2)	O19—S21—O22—C23	−63.7 (3)
O10—S8—N11—C12	43.0 (2)		

### 4-[(5-Methyl-1,2-oxazol-3-yl)sulfamoyl]anilinium methyl sulfate monohydrate Hydrogen-bond geometry (Å, º)

**Table:**

*D*—H···*A*	*D*—H	H···*A*	*D*···*A*	*D*—H···*A*
C3—H3···O24	0.93	2.65	3.353 (3)	133
C4—H4···O24^i^	0.93	2.54	3.469 (3)	177
C13—H13···O10	0.93	2.54	3.023 (3)	113
N1—H1*A*···O19^ii^	0.89	1.91	2.800 (3)	175
N1—H1*A*···S21^ii^	0.89	2.94	3.774 (2)	156
N1—H1*B*···O18	0.89	1.95	2.761 (3)	152
N1—H1*C*···O24	0.89	1.89	2.772 (3)	170
N11—H11···N16^iii^	0.86	2.07	2.912 (3)	167
C23—H23*B*···O20^iv^	0.96	2.65	3.506 (4)	149
O24—H24*A*···O10^v^	0.76 (3)	2.54 (3)	3.140 (3)	137 (3)
O24—H24*A*···O15^vi^	0.76 (3)	2.47 (3)	3.072 (3)	137 (3)
O24—H24*B*···O19^vii^	0.81 (3)	2.03 (3)	2.821 (3)	163 (3)

Symmetry codes: (i) −*x*, −*y*+1, −*z*+1; (ii) −*x*+1, −*y*, −*z*+1; (iii) −*x*+1, −*y*+1, −*z*; (iv) *x*+1, *y*, *z*; (v) −*x*+1, −*y*+1, −*z*+1; (vi) *x*−1, *y*, *z*+1; (vii) −*x*, −*y*, −*z*+1.
